# Hypothalamic Sirt1 in aging

**DOI:** 10.18632/aging.100627

**Published:** 2014-01-06

**Authors:** Akiko Satoh, Shin-ichiro Imai

**Affiliations:** Department of Developmental Biology Washington University School of Medicine St. Louis, MO 63110, USA

There is an old Japanese proverb saying, “After rain falls, the ground hardens.” This proverb illustrates what has happened to the research field on the importance of sirtuins in the regulation of aging and longevity. The Sir2 (*s*ilent *i*nformation *r*egulator 2) family of protein deacylases, now called sirtuins, have been shown to regulate aging and longevity in diverse model organisms including yeast, worms and flies. However, mice overexpressing Sirt1, the mammalian ortholog of Sir2, in the whole body failed to show life span extension (Herrenz et al. Nat. Commun., 2010, 1:3). Furthermore, it has been called into question whether increasing the dosage of Sir2 orthologs, sir-2.1 and dSir2, promotes life span extension in worms and flies (Burnett et al. Nature, 2011, 477:482). These results have brought considerable debates regarding the importance of sirtuins in aging/longevity control to the field of aging research. However, just as rain settles the soil, the entire field has acquired a much firmer foundation with the most recent and exciting findings (Kanfi et al. Nature, 2012, 483:218; Stumpferl et al. Genome Res., 2012, 22:1963-1973; Viswanathan and Guarente, Nature, 2011, 477:E1-2; Rizki et al. PLoS Genet., 2011, 7:e1002235; Schmeisser et al. Nat. Chem. Biol., 2013, 9:693-700; Mouchiroud et al. Cell, 2013, 154:430-441; Banerjee et al. Cell Rep., 2012, 2:1485-1491; Satoh et al. Cell Metab., 2013, 18:416).

Recently, we have demonstrated that increasing Sirt1 in the brain, particularly in the dorsomedial and lateral hypothalamic nuclei (DMH and LH, respectively), delays aging and extends life span (Satoh et al. Cell Metab., 2013, 18:416). Because our previous study showed that brain-specific *Sirt1-*overexpressing (BRASTO) transgenic mice significantly enhance physiological responses to dietary restriction (DR), we hypothesized that BRASTO mice could live longer than control wild-type mice under a regular chow-fed condition. Consistent with our expectation, BRASTO mice did show a fascinating extension of median and maximal life span in both males and females. For median life span, female BRASTO mice showed ~16% extension, whereas male BRASTO mice showed ~9% extension. Additionally, age-associated mortality and cancer-dependent death were significantly delayed in BRASTO mice, suggesting that the entire process of aging is significantly delayed in BRASTO mice. They also maintained their youthful physiology, including significantly higher physical activity, body temperature and oxygen consumption, and better quality of sleep, compared to age-matched controls. Interestingly, aged BRASTO skeletal muscle was found to specifically maintain youthful morphology and mitochondrial function during the process of aging. This intriguing phenotype was due to the enhancement of the sympathetic nervous tone followed by enhanced neural activity in the DMH and LH during the dark time in aged BRASTO mice; however, the mechanism by which the signal from the hypothalamus is specifically directed to skeletal muscle remains unknown. We also speculate that skeletal muscle stimulated by the sympathetic nervous system contributes to the delay in aging and possibly life span extension through the production of certain secreted factor(s). We are currently investigating this possibility.

In the DMH and LH, we found that Sirt1 cooperates with a novel molecular partner, Nk2 homeobox 1 (Nkx2-1). Sirt1 deacetylates two specific lysines in the homeodomain of Nkx2-1 and activates its function during the dark time. Sirt1 and Nkx2-1 together upregulate the expression of the orexin type 2 receptor (*Ox2r*) gene in the DMH and LH. Indeed, Sirt1 and Nkx2-1 highly colocalize in neurons of the DMH and LH. DMH/LH-specific stereotactic knockdown of *Sirt1*, *Nkx2-1*, or *Ox2r* confirmed the physiological importance of the Sirt1/Nkx2-1/Ox2r signaling pathway. This Sirt1/Nkx2-1/Ox2r-mediated pathway controls the sensitivity of a specific subset of DMH and LH neurons to orexin through the upregulation of *Ox2r* expression. Currently, the identity of these Sirt1/Nkx2-1 double-positive neurons is unknown. It will be of great importance to precisely characterize these Sirt1/Nkx2-1-double positive neurons to further understand the role of the DMH and LH in the regulation of mammalian aging/longevity.

A unique aspect of our study is the comparison between two independent lines of BRASTO mice (lines 1 and 10), which reveals a tight association between the neural activation in the DMH and LH, delay in aging, and life span extension in mice. In line 10 BRASTO mice, DMH/LH-predominant Sirt1 expression in the hypothalamus is crucial for enhanced neural activity in these two hypothalamic nuclei. This higher neural activity during the dark time in aged BRASTO mice compared to age-matched control mice resulted in multiple beneficial physiological phenotypes. On the other hand, line 1 BRASTO mice, which show increased Sirt1 expression throughout the hypothalamus, but not DMH/LH-predominantly, exhibited the total lack of life span extension and beneficial physiological phenotypes during the aging process. Indeed, the arcuate nucleus (Arc) in line 1 BRASTO mice exhibited much higher neural activity during the dark time compared to controls, whereas there was no enhancement of neural activity in the DMH and LH. It is possible that the enhancement of neural activity in the Arc could suppress the function of DMH and LH neurons in the regulation of aging and longevity in mammals. These results might also provide a reasonable explanation for why whole-body *Sirt1*-overexpression failed to promote life span extension. Further investigation will be required to examine the role of each hypothalamic nucleus in terms of mammalian aging/longevity control.

Our findings clearly reveal that the brain, the hypothalamus in particular, is one of the control centers for mammalian aging and longevity. Consistent with our finding, Zhang et al. have also demonstrated the importance of the hypothalamus in controlling mammalian aging/longevity (Zhang et al. Nature, 2013, 497:211). To further elucidate the physiological role of each hypothalamic nucleus in mammalian aging/longevity control, the following questions still remain: 1) Which hypothalamic nucleus is primarily susceptible to age-related deteriorations? 2) How are the levels of neuropeptides or neurotransmitters in each hypothalamic nucleus altered during the aging process? 3) Which particular physiological phenotypes regulated by the hypothalamus causally associate with mammalian longevity? The answers to these questions will further advance our understanding of the systemic regulation of mammalian aging and longevity and contribute to the development of effective anti-aging interventions. After many rainy days, we are happy to stand on firm ground and take the next step.

